# Chitosan composite scaffold combined with bone marrow-derived mesenchymal stem cells for bone regeneration: *in vitro* and *in vivo* evaluation

**DOI:** 10.18632/oncotarget.22917

**Published:** 2017-12-05

**Authors:** Shengqi Zang, Lei Zhu, Kefu Luo, Rui Mu, Feng Chen, Xiaocui Wei, Xiaodong Yan, Biyao Han, Xiaolei Shi, Qintao Wang, Lei Jin

**Affiliations:** ^1^ Department of Stomatology, Jinling Hospital, Medical School of Nanjing University, Nanjing, Jiangsu 210002, P.R. China; ^2^ Department of Prosthodontics, School of Stomatology, The Fourth Military Medical University, Xi’an, Shaanxi 710023, P.R. China; ^3^ Department of Stomatology, Urumqi General Hospital of PLA, Urumqi 830000, P.R. China; ^4^ Medical School of Southern Medical University, Guangzhou, Guangdong 510515, P.R. China; ^5^ Department of Periodontics, School of Stomatology, The Fourth Military Medical University, Xi’an, Shaanxi 710023, P.R. China

**Keywords:** chitosan, bovine-derived xenograft, bioscaffold, tissue engineering, calvarial defect

## Abstract

The study aimed to develop a chitosan (CS)-based scaffold for repairing calvarial bone defects. We fabricated composite scaffolds made of CS and bovine-derived xenograft (BDX), characterized their physicochemical properties including pore size and porosity, absorption, degradation, and compressive strength, compared their efficacy to support *in vitro* proliferation and differentiation of human jaw bone marrow-derived mesenchymal stem cells (hJBMMSCs), and evaluated their bone regeneration capacity in critical-size rat calvarial defects. The CS/BDX (mass ratio of 40:60) composite scaffold with porosity of 46.23% and pore size of 98.23 μm exhibited significantly enhanced compressive strength than the CS scaffold (59.33 ± 4.29 vs. 18.82 ± 2.49 Kpa). The CS/BDX (40:60) scaffold induced better cell attachment and promoted more osteogenic differentiation of hJBMMSCs than the CS scaffold. The CS/BDX (40:60) scaffold seeded with hJBMMSCs was the most effective in supporting new bone formation, as evidenced by better histomorphometry results, larger new bone area, and more obvious mature lamellar bone formation compared to other groups in rat calvarial defects 8 weeks after implantation. These results suggest that CS/BDX composite scaffold combining with hJBMMSCs has the potential for bone defect regeneration.

## INTRODUCTION

Bone grafting is beneficial in fixing bone defects caused by tumor resection, trauma, or other pathological conditions. Autogenous bone is considered the material of choice for bone grafting, but its application suffers from limited availability and increased risk of donor sites mortality. The unique morphology of the calvaria makes it even more difficult to obtain autogenous bone to reconstruct calvarial bone defects. Scaffold-based tissue engineering approach, involving the combination of a custom-made scaffold with cells/signaling molecules to direct tissue repair and restore tissue function, has emerged with great potential to be used in clinics to reconstruct calvarial bone defects [1]. However, this approach is challenged by issues such as the lack of ideal scaffold to implement and sustain necessary cell activities to regenerate tissue, and the lack of ideal cell type or insufficient quantities of ideal cells for clinical use.

To develop an ideal scaffold, a rational strategy is to fabricate a scaffold that can structurally, mechanically, and functionally mimic the extracellular matrix (ECM) of the target tissue. In natural bone tissue, ECM is a complex of organic-inorganic biocomposite, which mainly consists, in components, of type I collagen and hydroxyapatite [2].

Chitosan (CS) is a natural copolymer of (1→4)-2-acetamido-2-deoxy-β-D-glucan (N-acetyl D-glucosamine) and (1→4)-2-amino-2-deoxy-β-D-glucan (D-glucosamine) that is derived from chitin. The rationale behind the use of CS as a bone substitute is that CS is structurally similar to glycosaminoglycans, one of the primary components that connect with collagen fibers in ECM [3]. CS is widely used in wound healing due to its antimicrobial, biodegradable, and biocompatible properties. It also exhibits significant osteoconductivity by inducing proliferation of osteoblasts and promoting bone formation *in vitro* and *in vivo* [4–6]. In addition, CS has been found to be suitable to fabricate highly porous scaffolds with interconnected pores, which could mimic the native ECM of bone and allow for bone ingrowth to the implant sites. Together these properties support CS as a potential candidate for bone scaffold. However, CS has minimal osteoinductive property and a pure CS hydrogel/scaffold is weak in mechanical properties [7]. Compositing CS with hydroxyapatite cement seems to be a promising combination to overcome these weaknesses [8–10].

Bio-Oss (Geistlich AG, Wolhousen, Switzerland) is a commercially available form of hydroxyapatite that is widely used as a bone substitute for regenerative dentistry [11]. It is a porous bovine-derived xenograft (BDX) derived from cancellous bovine bone, with all organic components and pathogens being removed. The biocompatibility and osteoconductivity of Bio-Oss have been well documented, with most results favoring new bone formation and indicating that it can be partly replaced by the host tissues [11–13].

Mesenchymal stem cells (MSCs) have emerged as a promising cell source in scaffold-based tissue engineering, due to their inherent self-renewal and multipotent differentiation capacities [14]. Bone marrow-derived MSCs (BMMSCs) have the potential to differentiate into osteoblasts, chondrocytes, neurons, myoblasts, adipocytes, and fibroblasts, making them an attractive cell source for bone tissue engineering. They also have the advantages of easier isolation and expansion, less ethical concerns and lower risk of tumorigenesis as compared to embryonic stem cell [15]. Autogenous BMMSCs are commonly harvested from the iliac crest of donors. While frequently used, the procedure remains a concern because it could result in secondary damage and complications, and moreover, the harvested cells are limited in quantity [16]. Jaw BMMSCs (JBMMSCs), obtained from alveolar bone during the course of dental surgery (i.e., wisdom tooth extraction, crown lengthening surgery), appear to be an alternative with less invasive procedure and comparable differentiation and proliferative capacities [17].

In this study, to establish a flexible scaffold for sustained bone regeneration, a series of CS and Bio-Oss composite scaffolds were fabricated via the biomimetic mineralization process. These CS-based scaffolds were characterized morphologically, physicochemically and biologically to evaluate their efficacy for allowing JBMMSCs proliferation and differentiation *in vitro* and promoting new bone formation in rat calvarial defects *in vivo*.

## RESULTS

### Physicochemical characterization of CS-based scaffolds

Four types of composite scaffolds were fabricated, with mass ratios of CS to Bio-Oss (BDX) of 100:0, 70:30, 40:60, and 10:90. These scaffolds were evaluated and compared for their physicochemical characterization, including morphological structure, pore size and porosity, water absorption, degradation, and compressive strength.

**Figure 1:**
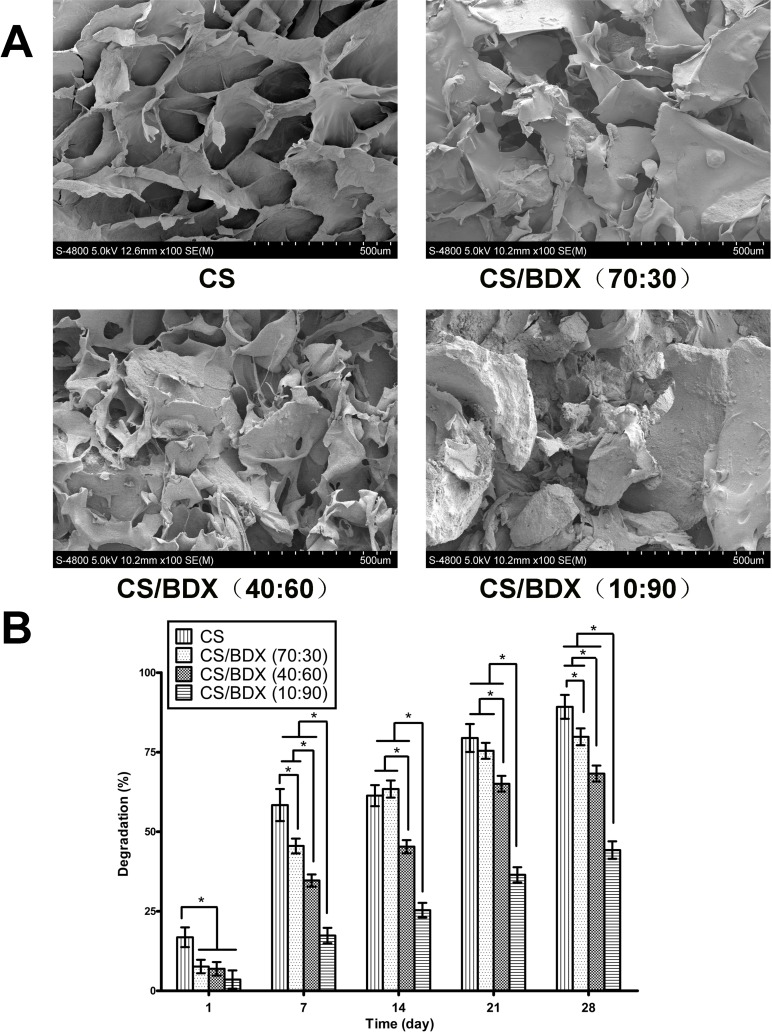
(**A**) SEM photomicrographs of the morphological structure of the scaffolds. (**B**) Degradation of the scaffolds over a 28-day time period. Four types of scaffolds fabricated at mass ratios of CS to BDX of 100:0, 70:30, 40:60, and 10:90, respectively, were evaluated. Data are shown as means ± SD with significance at *P < 0.05.

As shown in Figure 1A, a typical scanning electron microscopy (SEM) image of the CS scaffold had highly porous structure with interconnected pore channels. The morphology of CS-based scaffolds was affected by addition of BDX, being more irregular with increased content of BDX while retaining porous structure in all fabricated scaffolds.

Pore size and porosity of these scaffolds are present in Table 1, showing a correlation with the mass ratios of CS to BDX. The CS scaffold had the largest average pore diameter and the highest porosity level. The CS/BDX (70:30) and CS/BDX (40:60) scaffolds had significantly larger pore diameter and porosity than the CS/BDX (10:90) scaffold (*P* < 0.05), but did not differ significantly from each other (*P* > 0.05).

**Table 1:** Physicochemical properties of CS-based scaffolds fabricated with different mass ratio of CS to BDX

Scaffold type	Pore size (μm)	Porosity (%)	Water absorption (%)	Compressive strength (KPa)
CS	164.61 ± 39.32*	72.24 ± 8.13*	80.43 ± 6.54*	18.82 ± 2.49*
CS/BDX (70:30)	108.92 ± 22.36^#^	53.40 ± 7.19^#^	59.03 ± 7.37^#^	37.47 ± 5.78^#^
CS/BDX (40:60)	98.23 ± 25.53^#^	46.23 ± 9.89^#^	52.73 ± 5.16^#^	59.33 ± 4.29
CS/BDX (10:90)	62.15 ± 19.03	16.10 ± 6.30	19.70 ± 7.33	64.21 ± 5.20

*P < 0.05 compared with the CS/BDX (10:90), CS/BDX (40:60), and CS/BDX (70:30) groups.

^#^*P* < 0.05 compared with the CS/BDX (10:90) group.

As shown in Table 1, the ability of CS-based scaffolds to absorb water decreased with increased content of BDX. The CS scaffold absorbed the highest weight percentage of water (80.43%) after 24 hours of incubation with PBS, while the CS/BDX (10:90) scaffold only absorbed 19.70%. The CS/BDX (70:30) and CS/BDX (40:60) scaffolds exhibited little variations in water absorption (52.73% and 59.03%) in-between (*P* > 0.05).

Figure 1B shows the temporal changes of degradation in these CS-based scaffolds in the presence of lysozyme over a period of 28 days. In general, the CS scaffold had the fastest degradation rate, losing 16.84% of weight on the first day and almost all their mass (89.23%) by day 28. Increased content of BDX decreased degradation rate. The CS/BDX (70:30) and CS/BDX (40:60) scaffolds achieved 79.82% and 68.27% degradation on day 28, respectively, while the CS/BDX (10:90) scaffold showed only 44.23% degradation on the same day. A gradual incline in percentage of degradation occurred in all types of scaffolds over 28 days of incubation, with the most dramatic increase observed during the first 7 days and the increase almost reached plateau on day 21.

The compressive strength of scaffolds increased with increased content of BDX (Table 1). The CS scaffold had a significantly lower compressive strength than other scaffolds. The CS/BDX (10:90) scaffold had the highest compressive strength (64.21 ± 5.20 kPa), but it was not significantly different from that of the CS/BDX (40:60) scaffold (59.33 ± 4.29 kPa) (*P* > 0.05).

These results indicated that the CS/BDX (40:60) and CS/BDX (70:30) scaffolds were similar in pore size, porosity, absorption and degradation, but the former had much stronger compressive strength. As such, the CS/BDX (40:60) scaffold was chosen for the following *in vitro* and *in vivo* experiments, along with the CS and CS/BDX (10:90) scaffolds.

### Characterization of human JBMMSCs

Human JBMMSCs (hJBMMSCs), harvested from the alveolar bone of human subjects, were cultured in standard medium to evaluate MSC quality, using surface marker expression profiling and differentiation assays.

**Figure 2:**
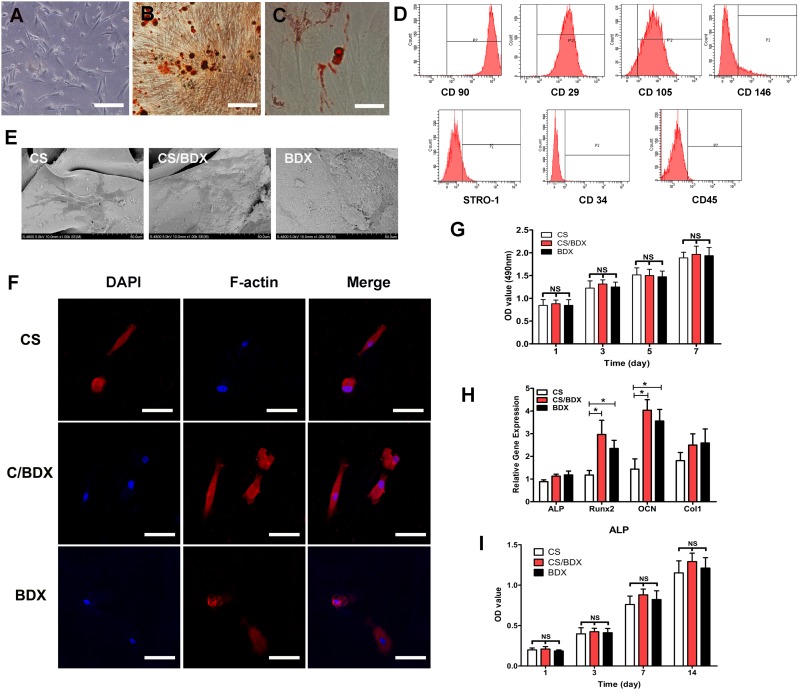
hJBMMSCs characterization and *in vitro* evaluation of hJBMMSCs on CS-based scaffolds (**A**) Morphology of hJBMMSCs at the first passage (scale bars = 50 mm). (**B**) Osteogenic differentiation of hJBMMSCs using Alizarin Red S staining (red color, scale bars = 50 mm). (**C**) Adipogenic differentiation of hJBMMSCs using Oil Red staining (red color, scale bars = 50 mm). (**D**) Flow cytometry profile of hJBMMSCs. (**E**) SEM photomicrographs of hJBMMSCs-seeded scaffolds after 3 days culture. (**F**) Confocal images of hJBMMSCs with dual staining of DAPI for nuclei (blue color) and phalloidin for F-actin (red color). (**G**) OD measurement of cell viability of hJBMMSCs on different scaffolds, cultured for 1 to 7 days. (**H**) Osteogenic gene expression of hJBMMSCs on different scaffolds, cultured in osteogenic induction medium at day 14. (**I**) OD measurement of ALP activity of hJBMMSCs on different scaffolds, cultured in osteogenic induction medium and standard medium at day 1, 3, 7, and 14. Data are shown as means ± SD with significance at *P < 0.05.

Cells in the primary culture displayed typical spindle-shape morphology of MSCs (Figure 2A). Cells in the fourth passage highly expressed MSC specific cell surface markers CD90 (99.2%), CD29 (95.8%), and CD105 (80.2%) and are positive for CD146 (12.9%) and STRO-1 (10.9%), and did not or low expressed hematopoietic markers CD34 (1.66%) and CD45 (0.39%) (Figure 2D). This expression profile indicated that MSC phenotype was retained in the fourth passages of hJBMMSCs.

After 4 weeks of osteogenic induction and 5 weeks of adipogenic induction, hJBMMSCs stained positive for mineral nodules (Figure 2B) and lipid droplets (Figure 2C), indicating that these cells retained their capacity to undergo multiple lineage differentiation into osteoblasts and adipocytes.

### *In vitro* evaluation of morphology, viability and differentiation of hJBMMSCs on scaffolds

Cell morphology of hJBMMSCs on the CS, CS/BDX (40:60), and CS/BDX (10:90) scaffolds was examined through microscopy based on DAPI and F-actin staining (Figure 2F) and confirmed by SEM (Figure 2E). After 3 days in culture, cells were flat and spread on the surface of these scaffolds, indicating good attachment of cells on scaffolds. Compared to the CS/BDX (10:90) scaffold, the CS/BDX (40:60) scaffold induced an obviously larger cell spreading area, while the CS scaffold led to reduced cell spreading area compared to the CS/BDX (10:90) scaffolds. Meanwhile, cells on the CS/BDX (40:60) scaffold were mainly polygonal shape, but those on the other two types of scaffolds were mainly two-polar spindle shape.

To examine the proliferation of hJBMMSCs on the CS, CS/BDX (40:60), and CS/BDX (10:90) scaffolds, variable cell density was determined by measuring optical density (OD) value. As shown in Figure 2G, at any measurement time point within 7 days of culture, cells on these three types of scaffolds exhibited comparable viability (*P* > 0.05). Being similar on all scaffolds, cell proliferation occurred since day 1 and increased over time with the plateau reached after 5 days of culture.

In this study, osteogenic differentiation potential of hJBMMSCs on scaffolds was assessed in term of gene expression of Runx2, ALP, Col1, and OCN, which are representative markers of different phases of osteogenic differentiation [18]. As shown in Figure 2H, these markers all expressed after 14 days of culture in osteogenic induction medium, whereas their expressions were different by scaffold types. Cells on the CS/BDX (40:60) scaffold conferred a 2.81-fold increase in OCN mRNA expression and a 2.52-fold increase in Runx2 mRNA expression compared to those on the CS scaffold, showing significant difference (*P* < 0.05). OCN and Runx2 expression levels in the CS/BDX (10:90) scaffold group were lower, but not significantly, than those in the CS/BDX (40:60) scaffold group. No significant difference in ALP and Col1 expression was observed among these three groups (*P* > 0.05).

Mineralization potential of hJBMMSCs on scaffolds was measured by alkaline phosphatase (ALP) assay. The results revealed that ALP activity kept increasing over the 14 days of culture (Figure 2I). As expected, ALP activity was significantly enhanced in cells cultured in osteogenic differentiation medium than those cultured in standard medium (*P* < 0.05). In consistent with the mRNA expression results, there was no significant difference in ALP activity between different scaffold types.

### *In vivo* evaluation of CS-based scaffolds for rat calvarial defect regeneration

To evaluate the ability of CS-based scaffolds to facilitate *in vivo* bone regeneration, the CS, CS/BDX (40:60), and CS/BDX (10:90) scaffolds, alone or seeded with hJBMMSCs, were implanted into critical-size calvarial bone defects created in rats. These rats were divided into 6 groups and each group consisted of 6 rats: CS/BDX+cell (CS/BDX (40:60) scaffold seeded with hJBMMSCs), CS/BDX (CS/BDX (40:60) scaffold), CS+cell (CS scaffold seeded with hJBMMSCs), CS (CS scaffold), BDX (CS/BDX (10:90) scaffold), and sham-surgery control. The surgical procedure is shown in Figure 6B. The procedure was well tolerated in all rats.

**Figure 3:**
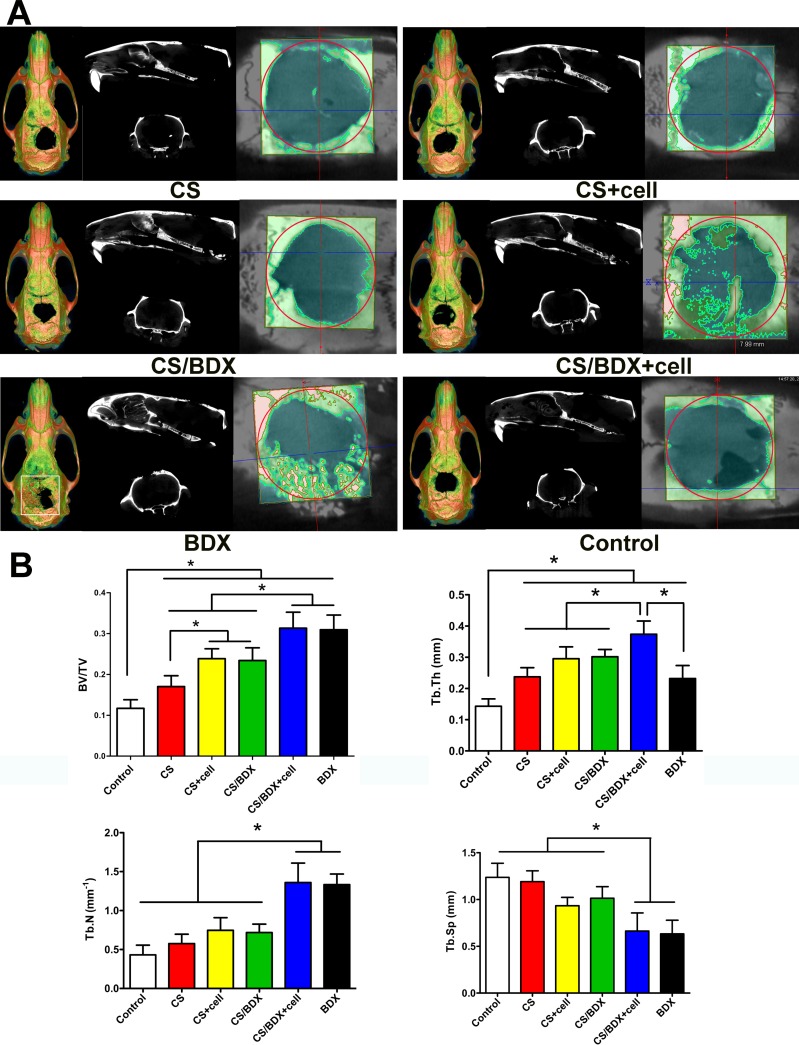
(**A**) Sagittal and coronal 2-dimension micro-CT scan images and 3-dimension reconstructed micro-CT images of critical-size circular calvarial defects 8 weeks after scaffold implantation. (**B**) Bone histomorphometry based on micro-CT images, including bone volume (BV/TV), trabecular thickness (Tb.Th), trabecular separation/spacing (Tb.Sp) and trabecular number (Tb.N). Data are shown as means ± SD with significance at *P < 0.05.

Micro-CT has been widely used to visualize structure and quantify density of newly formed bones [19]. In this study, micro-CT analysis was performed in scaffold/tissue constructs obtained from the defect sites 8 weeks post-surgery. Figure 3A shows the representative sagittal and coronal 2D graphs and 3D reconstructions. In the CS/BDX+cell group, newly formed bone mass originated from the bilateral margins of the calvarial defect and extended toward the center of the defect. New bone formation was less evident and mostly along the margin of the defect in the CS/BDX, CS+cell, and CS groups. In the BDX group, particles of residual Bio-Oss were substantial in the defect area and surrounded by newly formed bone. No obvious new bone formation was observed in the sham surgery control group.

**Figure 4:**
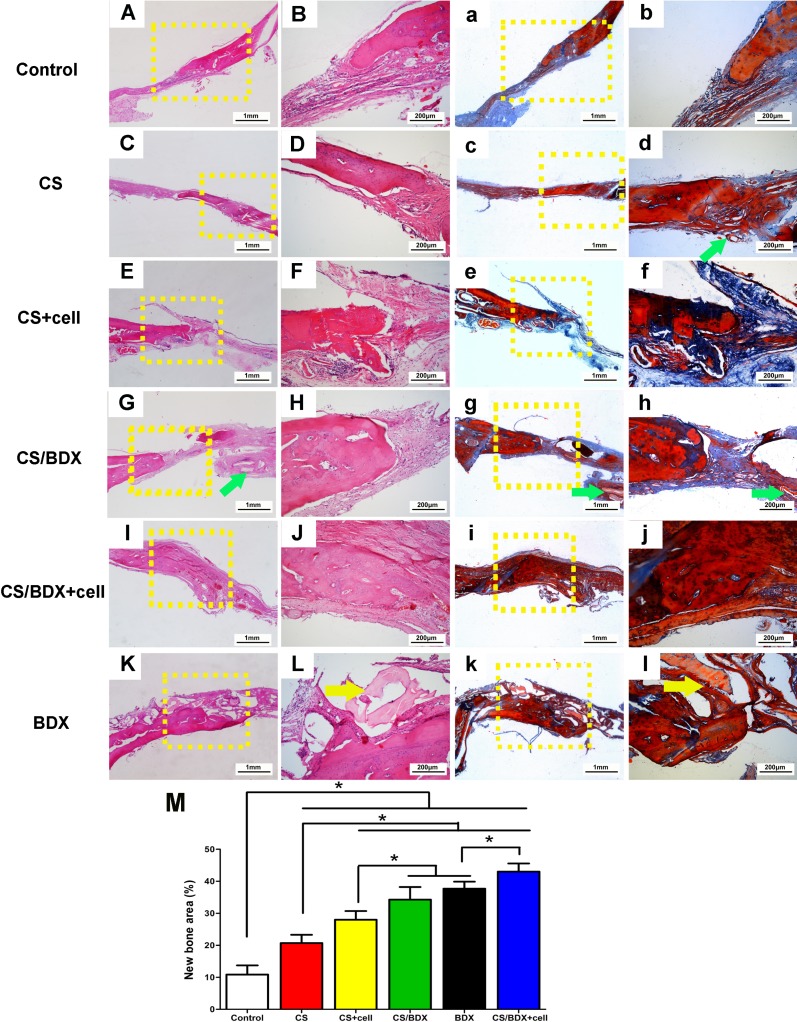
Histological photomicrographs (original magnification ×25 and ×100) with hematoxylin-eosin staining (**A**–**L**) and with Masson’s trichrome staining (**a**–**l**) of critical-size circular calvarial defects 8 weeks after scaffold implantation. Green arrow shows location of residual CS and yellow arrow shows location of residual BDX. (**M**) Histometric analysis of new bone area of defects 8 weeks after scaffold implantation. Data are shown as means ± SD with significance at *P < 0.05.

**Figure 5:**
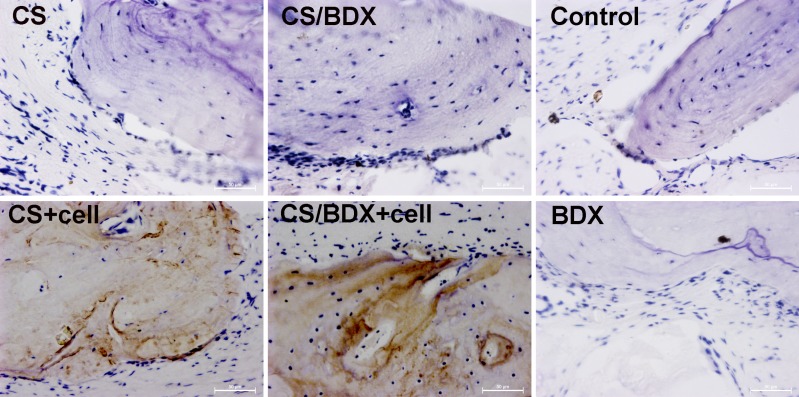
Immunohistochemical staining (brown stain, original magnification ×400) of osteocalcin in critical-size circular calvarial defects 8 weeks after scaffold implantation

**Figure 6:**
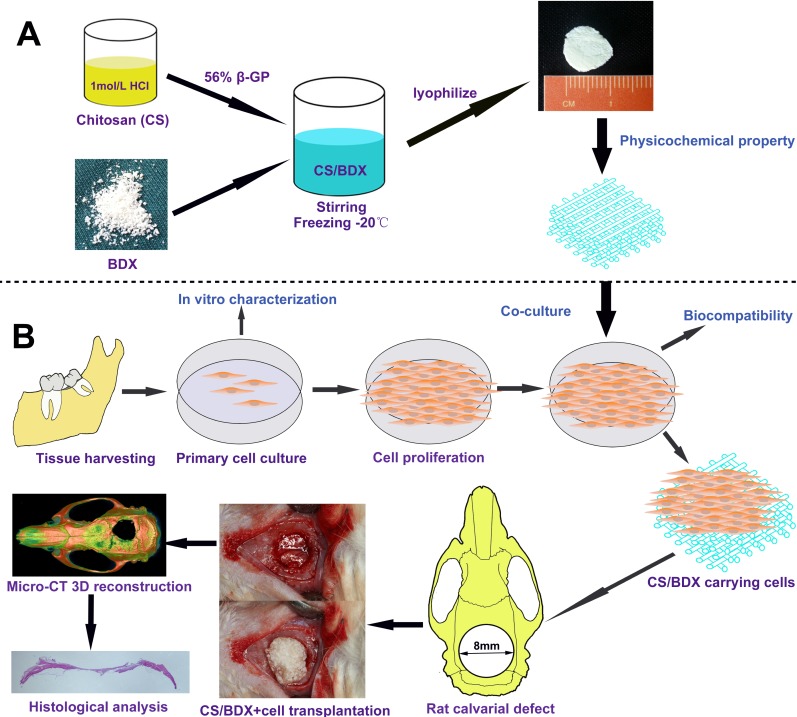
Schematic diagram of experiment design (**A**) Scaffold fabrication. (**B**) Cell harvest and culture, and scaffold implantation in rat model of calvarial defects.

Further quantitative analyses of the micro-CT images (Figure 3B) revealed that the CS/BDX+cell group had a significantly higher bone volume (BV/TV), trabecular thickness (Tb.Th), and trabecular number (Tb.N), and lower trabecular spacing (Tb.Sp) than the CS, CS+cell, CS/BDX, and control groups (*P* < 0.05, respectively). No significant differences in BV/TV, Tb.N and Tb.Sp were observed between the CS/BDX+cell and BDX groups. This is likely partly due to residual Bio-Oss particles in the BDX group (as observed in micro-CT image), which contains minerals with radiodensity similar to the natural bone.

The scaffold/tissue constructs were further evaluated for the histology. Hematoxylin-eosin staining of the CS/BDX+cell and CS/BDX groups showed marked new bone formation with typical structure of mature bone at the defect margin, and dense fibrous connective tissue in the center of defects (Figure 4G–4J). A scarce few CS particles embedded within newly formed bone were observed in the CS/BDX group (Figure 4G). New bone formation was less evident in the CS+cell group (Figure 4E and 4F), the CS group (Figure 4C and 4D) and the BDX group (Figure 4K and 4L). In the CS+cell group, CS particles appeared to be completely resorbed, whereas in the CS and BDX groups, CS and BDX particles scattered on the surface layer of fibrous connective tissue, respectively. In the sham control group, defects were filled with fibrous connective tissue along with minimal new bone formed from the peripheral host bone.

Masson’s trichrome staining showed collagen formation (blue color) in the defects. As illustrated in Figure 4, compared to the CS group, enhanced collagen deposition was observed in the CS+cell and CS/BDX groups. Moreover, a large amount of mature lamellar bone was observed in the CS/BDX+cell group.

Quantitative histological measurement for the new bone area fraction revealed that the CS/BDX+cell group had the highest percentage of newly formed bone, significantly above the levels of other groups (*P* < 0.05, respectively) (Figure 4M). The CS/BDX and BDX groups had similar new bone area percentage, and both were significantly higher than the CS+cell, CS, and control groups.

Osteocalcin is a marker for the mature osteoblast phenotype, specifically in lamellar bone. Osteocalcin staining can help to detect osteogenesis. The CS/BDX+cell and CS+cell groups showed numerous brown-violet nodules, indicating active osteoblast differentiation (Figure 5). In contrast, osteocalcin staining was not observed or obvious in other groups.

## DISCUSSION

CS and Bio-Oss are widely used bone substitutes, but each has their drawbacks. A major concern with using CS as the major component of a scaffold for bone tissue engineering is that it has relatively poor mechanical properties. Sufficient mechanical integrity is essential for a scaffold to function effectively during the bone defect repair process. In this study, CS and Bio-Oss composite scaffolds were fabricated through biomimetic mineralization. It was hypothesized that such an approach of assembling CS, an organic biomaterial naturally derived from ECM of bone tissue, with Bio-Oss, a bone-derived inorganic biomaterial, might generate a biomimetic microenvironment (scaffold) with improved mechanical and biological properties. Our results confirmed the hypothesis, showing a significantly increased compressive strength of the CS/BDX composite scaffold compared to the CS scaffold. This enhanced compressive strength might be explained by the possible interaction between the PO^3–^_4_ groups of BDX and the NH_3_^+^ groups of CS during fabrication process [8].

The architecture of scaffolds is another key consideration when designing a bone tissue scaffold. In the current study, a trade-off relationship between architecture properties and compressive strength was observed, showing higher porosity and larger pore size in scaffolds with lower compressive strength. It is well recognized that a high porosity or porous structure is essential for cell attachment, proliferation, and/or bone ingrowth in bone regeneration, but the optimal pore size remain a subject of debate. Scaffolds with pore sizes ranging between 20–1500 μm have been used in bone tissue engineering [20]. For example, scaffolds with a mean pore size of 100 μm were found to promote proliferation and enhance penetration of osteoblasts into the scaffolds *in vitro* [21], and scaffolds implanted *in vivo* with pore sizes of 100–135 μm induced significant bone growth [22], while in another study, a mean pore size of 325 μm was considered optimal for *in vitro* osteoblast adhesion and proliferation [20]. This wide range of recommended pore size may be due to complexity of bone regeneration process. In the study, except the CS/BDX (10:90) scaffold, the average pore sizes of CS-based scaffolds were between 98–165 μm, well around the recommended size range. After considering the trade-off between mechanical and architecture properties, the CS/BDX (40:60) scaffold was considered to be optimal. Further *in vitro* cell assays confirmed that compared to the CS and CS/BDX (10:90) scaffold, the CS/BDX (40:60) scaffold showed better cell attachment, and enhanced cell spreading and osteogenic differentiation of hJBMMSCs, indicating from morphological observation and upregulation of osteogenesis markers.

Biodegradability is a scaffold characteristic that is critical to the long-term performance of material-cell construct. Ideally, a scaffold should have an appropriate degradation rate that allows cells to produce body’s own ECM to replace the scaffold gradually. CS is bioabsorbable and is degraded *in vivo* by several proteases, mainly lysozyme, into non-toxic oligosaccharides of variable lengths [23], whereas biodegradability is a controversial issue with Bio-Oss, with some studies indicating hindering effect of persistence of Bio-Oss particles in bone remodeling [24, 25]. In this study, the CS scaffold had the fastest degradation rate and was degraded almost completely with the presence of lysozyme *in vitro* in 28 days. Adding BDX significantly reduced degradation of the scaffolds *in vitro*. This is in accordance with previous findings reporting reduced degradation rate after adding inorganic material to CS-based scaffolds, with mechanical strength maintained or even enhanced after long-term enzyme treatment [26, 27]. The *in vivo* data showed similar pattern, with BDX particles in the CS/BDX (10:90) scaffold group still occupying a solid portion of the defects 8 weeks after scaffold implantation. Of note is that BDX in the CS/BDX (40:60) scaffold seemed to be completely resorbed *in vivo*, regardless of whether the scaffolds were seeded with hJBMMSCs, though reason is unclear.

The success of bone regeneration at defect sites is directly related to the regenerative cells, which are characterized by their proliferation capacity and differentiation potential [14]. hJBMMSCs are easily harvested without any apparent side effects, making it good source of regenerative cells for bone tissue engineering. Our results showed that the harvested hJBMMSCs had a typical expression profile of MSCs, and moreover, were multipotent and differentiated into osteoblasts and adipocytes *in vitro*. Furthermore, our *in vivo* data confirmed that osteocalcin expression was evident in defects implanted with hJBMMSCs-seeded scaffolds, suggesting that hJBMMSCs survive and differentiate to osteoblast 8 weeks post-surgery in rats. In addition, no obvious immune reaction or inflammatory cells were observed or infiltrated in the surgical area. This finding is in agreement with previous studies, indicating that MSCs have low immunogenicity and immuneregulation ability, probably through inhibition of T lymphocyte and altered expression of MHC-I and NHC-II molecules [28–30]. As such, these results support hJBMMSCs’ use in combination with appropriate scaffolds in bone tissue engineering.

In addition to the regenerative cells, the microenvironment (scaffold) is instructive, providing a dynamic regulation of cellular behavior in bone regeneration, which involves cell recruitment and attachment, proliferation and differentiation [31]. Cell attachment directly affects cell proliferation, cellular signal transduction, and phenotype expression of cells [32]. Cell proliferation and differentiation are interrelated but distinct events, with the latter representing a crucial stage for success of regeneration. In the study, SEM and confocal results showed that the CS/BDX (40:60) scaffold exhibited better cell attachment. No significant difference was found between scaffolds in cell proliferation *in vitro*, but osteogenic differentiation of hJBMMSCs varied on different types of scaffolds. This result mirrors the findings of a previous study, which observed similar *in vitro* cell proliferation and different osteogenic differentiation performance between CS and CS/nano-hydroxyapatite/collagen Scaffolds [33]. In the study, the osteogenic differentiation of hJBMMSCs on scaffolds was profiled by gene expression of Runx2, ALP, Col, and OCN, and ALP activity. Runx2 is often referred to as the “master switch of osteogenic differentiation”, inducing osteogenic differentiation at the early stage and inhibiting it at the late stage [34]. Its gene expression is commonly analyzed in the early stage of osteogenic differentiation [35]. ALP is essential in the mineralization of the ECM with its expression usually indicating progression of osteogenic differentiation [36]. Col1 and OCN are constituents of the ECM and their expressions are used as mid and late markers of osteogenic differentiation, respectively [37]. Our results showed that hJBMMSCs induced significant early- and late- stage osteogenic differentiation (Runx2 and OCN) on the CS/BDX (40:60) scaffold compared to the CS scaffold, but did not lead to obvious difference in matrix mineralization (ALP expression and ALP activity) between these scaffolds *in vitro*. ALP activity showed that all CS-based scaffolds were osteoinductive.

An 8 mm diameter bone defect in rat is considered as a critical-size defect that would not heal spontaneously regardless how much time it is given to heal [38]. We used this criterion to create calvarial defects in our rat model and evaluated the combinative effect of CS-based scaffolds and hJBMMSCs on bone regeneration. The results were consistent with the findings in *in vitro* assays and demonstrated that the CS/BDX (40:60) scaffold seeded with hJBMMSCs was the most effective in supporting new bone formation 8 weeks post-surgery: the BV/TV, Tb.Th, and Tb.N values in the CS/BDX+cell group were notably higher than other groups based on micro-CT, the Tb.Sp value in the CS/BDX+cell group was much lower than other groups based on micro-CT, the total new bone area in the CS/BDX+cell group was significantly larger than other groups based on quantitative histology, and obvious mature lamellar bone formation was observed in the defects in the CS/BDX+cell group from Masson’s trichrome staining. This reinforcing effect of hJBMMSCs on CS-based scaffolds could be a direct effect in which hJBMMSCs differentiate into osteoblasts and initiate the cascade of new bone formation, or an indirect effect in which hJBMMSCs act by secreting regulatory growth factors to create favorable microenvironment for bone regeneration [39]. Our osteocalcin expression results support the former assumption by observing differentiated osteoblasts lied scattered throughout the defects implanted with cell-seeded scaffolds. The results of *in vivo* evaluation should be interpreted with caution. Although our results support that the CS/BDX scaffold combined with hJBMMSCs could promote bone regeneration in defects 8 weeks after implantation, there is no enough evidence to favor long-term effects of this composite scaffold in inducing bone healing and formation. As such, future long-term *in vivo* studies are warranted to validate the findings of current study.

## CONCLUSIONS

This study found that the CS/BDX (40:60) composite scaffold had improved physicochemical properties, and supported hJBMMSCs proliferation and differentiation *in vitro*. Furthermore, *in vivo* bone regeneration capacity of hJBMMSCs-seeded CS/BDX (40:60) scaffold, in comparison with CS scaffold and CS/BDX (10:90) scaffold, was assessed to determine the potential application of such a combination of scaffold and MSCs. Significantly, our results showed enhanced new bone formation in critical-size calvarial defects in rats implanted with hJBMMSCs-seeded CS/BDX (40:60) scaffold. Given that the CS/BDX (40:60) scaffold can be readily and flexibly fabricated as needed clinically and have relative sound mechanical strength, we propose that it could be a promising composite biomaterial for bone tissue engineering and such a combination of CS-based scaffolds with hJBMMSCs is a candidate approach for fixing bone defects.

## MATERIALS AND METHODS

### Scaffold fabrication

Figure 6A shows the schematic diagram of scaffold fabrication process. A 2% (w/v) CS solution was prepared by adding 200 mg CS (median molecular weight, degree of deacetylation of 75–85%; Sigma-Aldrich, MO, USA) to 9 mL hydrochloric acid (0.1 mol/L), and stirring until completely dissolved. A glycerol phosphate (GP) solution was prepared by dissolving 560 mg β-GP (E. Merck, Germany) in 1 mL distilled water and sterilizing with filtration. Before use, both solutions were chilled in an ice bath for 15 min to avoid gelation. Then a 1 mL GP solution was added dropwise to a 9 mL CS solution with continuous stirring to form a clear homogeneous CS/GP solution. To fabricate four types of CS/BDX scaffolds, the CS/GP solution and BDX (Bio-Oss Geistlich AG, Wolhousen, Switzerland) were mixed at mass ratios of 100:0, 7:30, 40:60, and 10:90, stirred for 10 min on ice, frozen at –20°C overnight, and then lyophilized for 24 h. The fabricated scaffolds were then sterilized with γ-irradiation from a ^60^Co source at 15 kGy, as described previously [40].

### Scaffold characterization

Porosity and pore size: The porosity was determined using the Archimedes’ Principle according to Miranda *et al.* [41]. Dried standard-size scaffolds were immersed in ethanol at room temperature and porosity (in percentage) was calculated using the following equation:

$$Porosity\left(\%\right)=\frac{\left(W_{2}-W_{1}\right)}{\rho V_{1}}\times 100\%$$

where *W*_*1*_ is the weight of dried scaffold (g), *W*_*2*_ is the weight of scaffold (g) after immersed in ethanol for 24 h, is the density of the ethanol, and *V*_*1*_ is the volume of dried standard-size scaffold (cm3).

The pore size was determined by SEM (S-4800, Hitachi, Japan). Images of scaffolds coated with a gold-palladium layer were captured from multiple sites and mean pore diameters were calculated.

Water absorption: The experiment was repeated in triplicate using the method described previously [42]. Dried scaffolds were weighted (*W*_*0*_) and fully rehydrated with phosphate buffered saline (PBS, pH=7.4) at 37°C for 24 h. The scaffolds were then blotted dry on filter paper and reweighted (*W*_*s*_). Water absorption (in percentage) was calculated using the following equation:

$$Water\;absorption\left(\%\right)=\frac{W_{s}-W_{0}}{W_{0}}\times 100\%$$

Degradation: Cylindrical scaffolds (1 cm in diameter, 2 mm in height) were incubated in PBS (pH=7.4) containing 1.5 μg/ml chicken egg white lysozyme (Wolsen Biotechnology Co. Ltd., China). The concentration of lysozyme was chosen to mimic the level in human serum [43]. The incubation lasted 28 days at 37°C and the medium was refreshed daily to ensure continuous enzyme activity [44]. Dry weights of the scaffolds were measured on day 1, 7, 14, 21, and 28 of the incubation. Degradation was determined as the percentage of weight loss using the following equation:

$$Weight\;loss\left(\%\right)=\frac{W_{L}-W_{0}}{W_{0}}\times 100\%$$

where *W*_*0*_ is the initial weight of scaffolds and *W*_*L*_ is the weight after incubation.

Compressive strength: Compressive strength test was performed using texture analyzer (TMS-Pro, Sterling, VA, USA). A vertical load was applied to the flat surface of cylindrical scaffolds (10 mm in diameter, 20 mm in height) and the scaffolds were compressed at a speed of 1 mm/min.

### JBMMSCs isolation and culture

Human JBMMSCs (hJBMMSCs) were obtained from the alveolar bone of human subjects according to the method as previously described [45]. The protocol was approved by the Ethical Committee of Jinling Hospital, Medical School of Nanjing University, China. Informed consent was obtained from each human subject. Briefly, bone marrow was aspirated from the trabecular bone of 6 healthy donors (14–28 years old) without gingivitis or periodontitis during impacted wisdom teeth extraction procedure. The bone marrow was then cultured in Dulbecco’s Modified Eagle Medium (DMEM) supplemented with 10% fetal bovine serum (FBS, Gibco/Invitrogen, CA, USA), 50 μg/mL streptomycin sulfate, and 100 U/mL penicillin. The third to fifth passage cells were used for the following experiments.

### Flow cytometric analysis

Before seeded on scaffolds, hJBMMSCs underwent flow cytometric analysis to characterize the immunophenotypic profile using CD29, CD34, CD45, CD90, CD105, CD146 and STRO-1. The fourth-passage cells (2 cells) were fixed with 4% paraformaldehyde for 15 min, resuspended in PBS with 3% FBS, incubated with saturating concentrations (1:100 dilution) of primary antibodies (BD Bioscience, CA, USA) for 1 h at 4°C in the dark, and analyzed on a FACS Vantage cell sorter (Becton Dickinson, CA, USA) using FlowJo software (version 7.6.5, Tree Star Inc., OR, USA).

### Multipotent differentiation assay

Before seeded on scaffolds, hJBMMSCs were examined for their capacity to differentiate into either adipocytes or osteocytes. Osteogenesis differentiation was induced by incubating cells at a density of 4 × 10^3^ cells/cm^2^ in osteogenic medium containing 10 mM β-glycerophosphate, 10 nM dexamethasone, 50 mg/mL ascorbate phosphate (all from Sigma-Aldrich, MO, USA), and 10% FBS for 4 weeks. Mineral deposits indicating osteogenesis differentiation were stained with 40 mM Alizarin Red S (Sigma-Aldrich, MO, USA). Adipogenesis differentiation was induced by incubating cells at a density of 4 × 10^3^ cells/cm^2^ in adipogenic medium containing 1 mM dexamethasone, 0.2 mM indomethacin, 0.01 mg/mL insulin and 0.5 mM isobutyl-methylxanthine (all from Sigma-Aldrich, MO, USA), and 10% FBS for 5 weeks. Lipid vacuoles indicating adipogenesis differentiation were stained with Oil Red staining (Sigma-Aldrich, MO, USA).

### Cell morphology and viability on scaffolds

hJBMMSCs were seeded on scaffolds in wells of 48-well plates at a density of 1 × 10^6^ cells/well. After 3 days of culture, the cell-seeded scaffolds were fixed with 0.25% glutaraldehyde for 24 h, washed with PBS, and then dehydrated with a gradient ethanol. After dry, scaffolds were coated with a gold–palladium layer and cells on scaffolds were imaged under SEM. To visualize the actin cytoskeleton under a confocal laser scanning microscope (FV1000, Olympus, Japan), after permeabilized with PBS containing 0.1% Triton X-100, cells on scaffolds were stained with Rhodamine-conjugated Phalloidin solution (50 μg/ml in PBS) for 40 min at room temperature and then cell nuclei were counterstained with 4,6-diamidino-2-phenylindole (DAPI, 1 μg/ml, Roche, IN, USA).

Cell viability in hJBMMSCs-seeded scaffolds was assessed using a Cell Counting KIT-8 kit (CCK-8, Beyotime Biotechnology, China). The cell-seeded scaffolds were cultured in DMEM with 10% FBS for 1 to 7 days and then incubated in CCK-8 working solution at 37°C for 4 h. The absorbance was measured at 450 nm.

### RT-PCR for osteogenic-specific gene expression

Gene expression of osteogenic-specific genes ALP, Runx2, OCN, and Col1 were compared among different scaffold groups (*n* = 3, for each group). hJBMMSCs were seeded on scaffolds and incubated in osteogenic differentiation medium for 14 days. Cells were then removed from culture and total cellular RNA was isolated by lysis in TRIzol (Invitrogen, CA, USA). RT-PCR was performed with SYBR Premix Ex TaqI restriction enzyme (ITli RNaseH plus, TakaRa, Japan) on an ABI 7500HT Fast Real-Time PCR system (Applied Biosystems, NY, USA). The PCR conditions were 95°C for 30 s followed by 40 cycles of 95°C for 5 s and 60°C for 34 s. The primer sequences (synthesized by Sangon Biotech Co., Ltd., Shanghai, China) were: Runx-2 (forward: 5′-CACTGGCGCTGCAACAAGA-3′ and reverse: 5′-CACTGGCGCTGCAACAAGA-3′), OCN (forward: 5′-CCCAGGCGCTACCTGTATCAA-3′ and reverse: 5′-GGTCAGCCAACTCGTCACAGTC-3′), COL I (forward: 5′-GCAAGGTGTTGTGCGATGA-3′ and reverse: 5′-TGGTCGGTGGGTGACTCTG-3′), ALP (forward: 5′-CCTTGTAGCCAGGCCCATTG-3′ and reverse: 5′-GGACCATTCCCACGTCTTCAC-3′), and the housekeeping gene GAPDH (forward: 5′-GCACCGTCAAGGCTGAGAAC-3′ and reverse: 5′-TGGTGAAGACGCCAGTGGA-3′). Data were analyzed for relative expression using the 2^–∆∆Ct^ method and normalized to GAPDH.

### Alkaline phosphatase (ALP) activity

ALP assay was performed to assess the mineralization activity of hJBMMSCs cultured on scaffolds (*n* = 3, for each group). hJBMMSCs were seeded on scaffolds and incubated in standard medium and osteogenic differentiation medium, respectively, at 37°C for 1, 3, 7, and 14 days. Culture medium was refreshed at 3-day intervals. After culture, cell-seeded scaffolds were rinsed with PBS and cell lysate was obtained by incubating the scaffolds with 0.2% Triton X-100 overnight. ALP activity was evaluated using an ALP kit (Jiancheng Bioengineering Institute, China) according to manufacturer’s instructions. The OD was measured at 520 nm with an automatic microplate reader (Bio-Tek, VT, USA).

### Animal procedures

All animal studies were performed in accordance with the guidelines for experimental animals at Nanjing University and the Guideline for the Care and Use of Laboratory Animals from the National Institutes of Health, USA. The protocol was approved by the Ethics Committee of Jinling Hospital, Nanjing University. Thirty six 8-weeks-old female Sprague-Dawley rats (250–300 g) were obtained from the Experimental Animal Center of Jinling Hospital, Nanjing University. The animals were maintained with a 12-hour day/night cycle, an ambient temperature of 21°C, and ad libitum access to water and pellet diet.

The rats were anesthetized by intramuscular injection (50 mg/kg body weight) of 1% pentobarbital sodium, along with local anaesthetic lidocaine. An incision of approximately 2 cm was made along the sagittal plane on the cranium and a full thickness flap was reflected to expose the calvarial bone. Critical-size circular calvarial defects (diameter, 8.0 mm) were created in the cranium using a saline cooled trephine drill (Figure 6B). After removal of the full thickness of the cranial bone in the defect, thirty-six rats were randomly assigned, in equal numbers, to the followed 6 groups and implanted with different scaffolds: CS/BDX (40:60) scaffold, CS/BDX (40:60) scaffold seeded with 1 × 10^7^ cells/mL hJBMMSCs, CS scaffold, CS scaffold seeded with 1 × 10^7^ cells/mL hJBMMSCs, CS/BDX (10:90) scaffold, and a sham-surgery control group. The periosteum and skin were then closed and sutured with 4–0 nonresorbable suture material. The sutures were removed 12 days later. All rats were sacrificed 8 weeks after surgery. The scaffolds and surrounding tissue were excised and fixed in a 10% neutral buffered formalin solution for 14 days.

### Microcomputed tomography (micro-CT) analysis

Excised samples in the cranium were scanned with micro-CT (Siemens Inveon, Germany) to evaluate new bone formation within the implant sites. The scan was at energy of 80 kV and 500 μA, a current of 145 mA, and an isotropic voxel size of 27 μm. After 3D reconstruction, quantitative analyses of bone volume/total volume (BV/TV), trabecular thickness (Tb.Th), trabecular separation/spacing (Tb.Sp) and trabecular number (Tb.N) in the same region of interest within defects were performed using Inveon Research Workplace 2.2 [46].

### Histological and immunohistochemical analysis

Each cranium was decalcified in 10% EDTA for 30 days and embedded in paraffin. After hardening, longitudinal sections were cut into 5–7 µm slices and stained with hematoxylin-eosin and Masson’s trichrome staining methods. After conventional microscopic examination, newly formed bone was examined under light microscopy and the area of new bone formation was quantitatively evaluated using Image-Pro Plus System (Media Cybernetics, MD, USA).

Immunohistochemical analysis of osteocalcin protein expression was performed using a Histostain-Plus IHC Kit (Jiancheng Bioengineering Institute, China) according to the manufacturer’s instruction. In brief, the 5–7 µm slices were probed with mouse anti-rat monoclonal OCN primary antibody (10 µg/ml, Abcam) overnight at 4°C, then incubated with biotinylated anti-mouse IgG for 20 min, and exposed to horseradish peroxidase labeled streptavidin for 30 min. The presence of antibody-antigen complexes was visualized with diaminobenzidine and counterstained with hematoxylin for 5 min. The stained slides were photographed digitally under a microscope (DMI6000, Leica, Germany).

### Statistical analysis

The data were expressed as means ± standard deviation (SD). The one-way analysis of variance (ANOVA) and Student-Newman-Keuls post hoc test were used to calculate statistical significances among groups. A *P* value less than 0.05 was considered statistically significant. All statistical analyses were performed using SPSS 13.0 statistical software (SPSS, IL, USA).

## Abbreviations

ALP: Alkaline phosphatase
ANOVA: analysis of variance
BDX: bovine-derived xenograft
BMMSC: bone marrow-derived mesenchymal stem cell
CS: chitosan
DMEM: Dulbecco’s modified eagle medium
ECM: extracellular matrix
FBS: fetal bovine serum
GP: glycerol phosphate
MSC: mesenchymal stem cell
micro-CT: Microcomputed tomography
OD: optical density
PBS: phosphate buffered saline
SD: standard deviation
SEM: scanning electron microscopy
